# Supplementary material for: Causal Relationship between Aging and Anorexia Nervosa: A White-Matter-Microstructure-Mediated Mendelian Randomization Analysis
Source: Biomedicines. 2024 Aug 16;12(8):1874. doi: 10.3390/biomedicines12081874 (PMC11351342; doi:10.3390/biomedicines12081874)
Supplement: Supplementary file 1 [file biomedicines-12-01874-s001.zip › Supplementary Figure/Figure S4/Singe SNP plot of MR analysis for TL to ubm-b-1742.pdf]

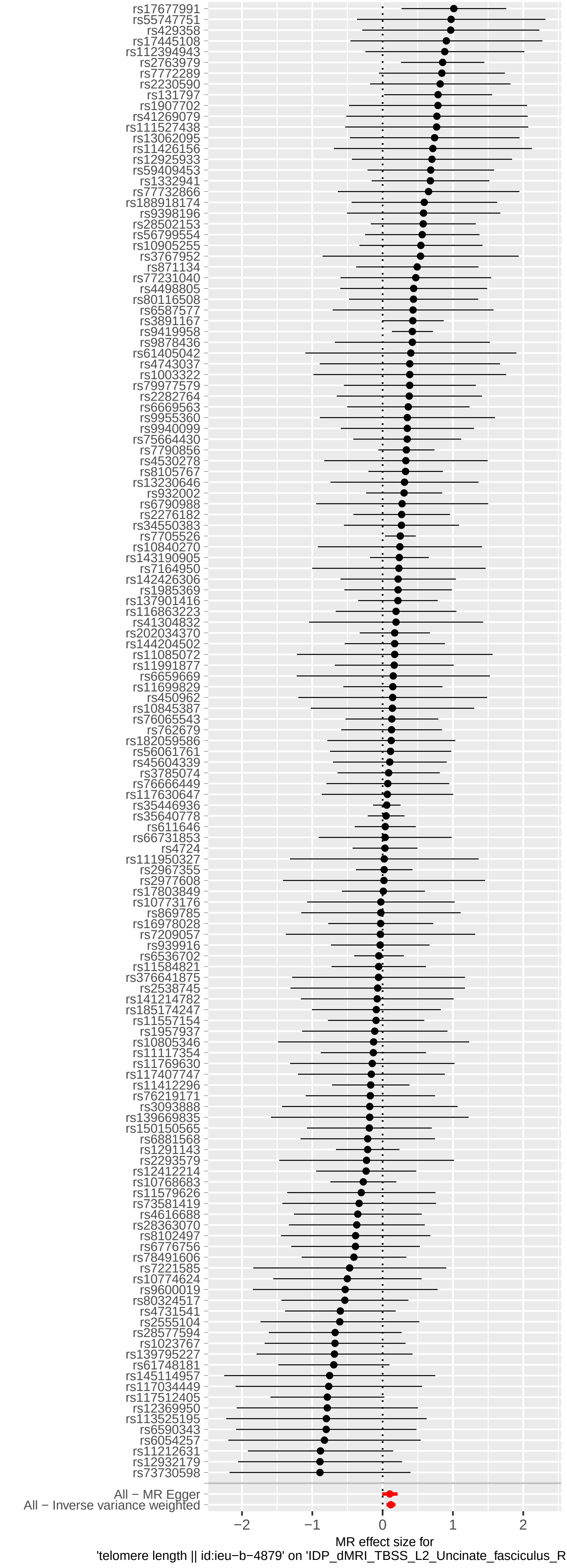
rs17677991
rs55747751
rs429358
rs17445108
rs112394943
rs2763979
rs7772289
rs2230590
rs131797
rs1907702
rs41269079
rs111527438
rs13062095
rs11426156
rs12925933
rs59409453
rs1332941
rs77732866
rs188918174
rs9398196
rs28502153
rs56799554
rs10905255
rs3767952
rs871134
rs77231040
rs4498805
rs80116508
rs6587577
rs3891167
rs9419958
rs9878436
rs61405042
rs4743037
rs1003322
rs79977579
rs2282764
rs6669563
rs9955360
rs9940099
rs75664430
rs7790856
rs4530278
rs8105767
rs13230646
rs932002
rs6790988
rs2276182
rs34550383
rs7705526
rs10840270
rs143190905
rs7164950
rs142426306
rs1985369
rs137901416
rs116863223
rs41304832
rs202034370
rs144204502
rs11085072
rs11991877
rs6659669
rs11699829
rs450962
rs10845387
rs76065543
rs762679
rs182059586
rs56061761
rs45604339
rs3785074
rs76666449
rs117630647
rs35446936
rs35640778
rs611646
rs66731853
rs4724
rs111950327
rs2967355
rs2977608
rs17803849
rs10773176
rs869785
rs16978028
rs7209057
rs939916
rs6536702
rs11584821
rs376641875
rs2538745
rs141214782
rs185174247
rs11557154
rs1957937
rs10805346
rs11117354
rs11769630
rs117407747
rs11412296
rs76219171
rs3093888
rs139669835
rs150150565
rs6881568
rs1291143
rs2293579
rs12412214
rs10768683
rs11579626
rs73581419
rs4616688
rs28363070
rs8102497
rs6776756
rs78491606
rs7221585
rs10774624
rs9600019
rs80324517
rs4731541
rs2555104
rs28577594
rs1023767
rs139795227
rs61748181
rs145114957
rs117034449
rs117512405
rs12369950
rs113525195
rs6590343
rs6054257
rs11212631
rs12932179
rs73730598
All − MR Egger
All − Inverse variance weighted
−2
−1
0
1
2
MR effect size for
'telomere length || id:ieu−b−4879' on 'IDP_dMRI_TBSS_L2_Uncinate_fasciculus_R
